# Short-Term Habituation of Auditory N1 in Spoken Word-Forms Is Modulated by Phonological Information

**DOI:** 10.3390/brainsci12101279

**Published:** 2022-09-22

**Authors:** Jinxing Yue, Peng Wang, Jiayin Li, Zhipeng Li, Xia Liang, Yifei He

**Affiliations:** 1 Laboratory for Cognitive and Social Neuroscience, School of Management, Harbin Institute of Technology, Fayuan Street 13, Harbin 150001, China; 2 Brain Networks Group, Max Planck Institute for Human Cognitive and Brain Sciences, Stephanstrasse 1A, 04103 Leipzig, Germany; 3 Institute of Psychology, University of Regensburg, Sendrastrasse 1, 93055 Regensburg, Germany; 4 School of International Studies, Harbin Institute of Technology, West Dazhi Street 92, Harbin 150001, China; 5 Centre for Space Environment and Physical Sciences, Harbin Institute of Technology, West Dazhi Street 92, Harbin 150001, China; 6 Department of Psychiatry and Psychotherapy, Philipps University Marburg, Rudolf Bultmann-Strasse 8, 35039 Marburg, Germany

**Keywords:** N1, habituation, auditory evoked potential (AEP), event-related potential (ERP), speech, auditory, phonology

## Abstract

Short-term auditory habituation is typically reflected by decreased but recoverable amplitudes of the N1 component of event-related potentials to repeated stimuli. It remains less well understood whether and how N1 habituation is modulated by the human cognition. The current study aims to further test for the potential modulatory roles of phonological information carried by spoken word-forms. Two phonological variables, namely lexicality (real versus pseudoword-form) and usage frequency (high versus low frequency), are considered and combined factorially, yielding four types of monosyllabic Mandarin spoken word-forms. Each type consists of 10 items (i.e., word-forms). The stimuli were passively presented to native Mandarin speakers in trains of five (S1–S5), while their EEG was recorded. The peak amplitudes of N1 to the same type of speech stimuli were calculated for each position by averaging the trains extracted from the EEG recording. Then, the N1 habituation was quantified for the two electrodes of interest (C3 and C4) in each repetitive presentation position (S2–S5). The results showed that the N1 habituation in low-frequency pseudo word-forms was consistently greater than in low-frequency real word-forms and high-frequency pseudo word-forms, respectively, at the fourth presentation (S4). The results suggest the first evidence that different types of phonological information (i.e., lexicality and usage frequency) modulate N1 habituation, interactively. Sensory filtering is proposed as a candidate mechanism for mediating between the processing of phonological information and the short-term habituation of auditory N1.

## 1. Introduction

Neural responses to repeated stimulations tend to decrease. This kind of adaptive neural response pattern has been attributed to mechanisms such as repetition suppression [1], habituation [2], sensory gating [3], or, more generally, neural adaptation [4,5]. It is considered as a basic manifestation of learning, which forms the foundation for developing more complicated neural plasticity [6,7,8].

Here, we focus on a special type of repetition-induced neural decrement, that is, the short-term habituation of N1 component of auditory evoked potentials. While this phenomenon has traditionally been approached as a physiological mechanism [9], some research has reported that the short-term habituation of N1 responses to auditory stimuli can also reflect a certain degree of cognitive tuning [10]. To further this issue, in the present study, we investigate whether and how this physiological mechanism is modulated by the phonological information as represented by spoken word-forms during passive speech perception. By manipulating two phonologically factors, namely lexicality and usage frequency, factorially, in Mandarin monosyllabic word-forms, we conduct a passive short-term habituation experiment, in which each train of five repeatedly presents the same word-form. We find the first evidence that the short-term auditory habituation of the evoked N1 response is interactively modulated by different types of phonological information.

## 2. Literature Review

### 2.1. Short-Term Auditory Habituation

In the auditory domain, when repeated stimuli are presented in a train consisting of a few presentation positions, separated by constant but brief interstimulus intervals (e.g., 500 ms), they usually elicit decreased amplitudes of evoked responses. More importantly, the decrement of response can recover to the original level at the initial presentation position of another train, given a 4 to 10 s stimulus-free inter-train interval [11,12], and thus, it is termed as short-term auditory habituation [13,14].

Short-term auditory habituation is usually recorded by neurophysiological measures, such as electroencephalography (EEG) and magnetoencephalography (MEG) at high temporal resolution, which offer millisecond accuracy. Short-term auditory habituation is already observable at the earliest latencies, roughly between 50 and 200 ms post-stimulus onset, as indexed by decreased amplitudes of auditory-evoked brain potentials (AEPs) of P1, N1, and P2 or their magnetic equivalents (auditory-evoked field components, AEFs) (e.g., for studies with EEG, see [15,16,17]; for studies with MEG, see [10,18]). The three successive components of auditory AEPs (also called a P1-N1-P2 complex) are assumed to reflect a summed synchronous firing of auditory neurons, indicating obligatory auditory responses, during pre-attentive processing [19]. Moreover, it is believed that auditory N1, recorded around the vertex, reflects the most typical short-term auditory habituation [11,12,13,18,20].

A physiological account for the short-term auditory habituation of auditory N1 (N1 habituation for simplicity hereafter) is neural refractoriness, which is a *pure*, bottom–up adaptation mechanism [9,18,21]. In detail, when the neurons respond to quickly repeated stimuli, they are possibly subjected to the depletion of recyclable neurotransmitters to generate a postsynaptic response with the same magnitude as that to the first stimulus [22,23]. According to this account, the N1 habituations to different auditory stimuli should be alike if they have similar acoustic parameters because of the highly overlapping neural representations corresponding to these stimuli (also *cf*., [24,25]).

### 2.2. N1 Habituation Modulated by Cognition

Apart from this refractory account, there are also findings suggesting top–down influences on the repetition-related decrement of early auditory evoked responses, originated from cognitive functions such as selective attention [26] and expectation [27,28,29]. For example, Todorovic et al. (2011) [29] found that compared to expected stimulus repetitions, unexpected repetitions of a pure tone led to stronger neurophysiological responses from 100 ms post-stimulus onset.

Furthermore, a few studies have suggested that N1 habituation is sensitive to the distinction between speech and non-speech stimuli, even if the basic acoustic features are matched (e.g., [10,14]). For example, by using MEG, Teismann and colleagues (2004) [10] revealed differential N1m habituation patterns to repeated speech sounds and tones. Their data showed that repeated vowels induced greater N1m decrement in the right hemisphere than in the left hemisphere, whereas no difference between the two hemispheres was observed in the repetitions of a tone.

More recently, in an experiment with a short-term habituation design, divergent patterns of N1 attenuation, recorded around the vertex, were found between the repetitions of vowels and those of the spectrally rotated versions of the vowels, in which the former elicited smaller N1 habituation than the latter did. Since the two types of stimuli are strictly controlled for acoustic variables, a plausible account for this result is that N1 habituation is impacted by acoustic categorisation. That is, in order to distinguish between a vowel and a bunch of acoustic signals which cannot be categorised into vowels but still sound like speech, the brain relies on more abstract, high-order neural representations of the category of vowels. Taking all the above previous findings together, an interim conclusion can be made that in the auditory domain, the short-term habituation of N1 can be modulated by high-order cognitive functions related to auditory/speech perception.

### 2.3. N1 Habituation and Phonological Information

Based on the literature of an interplay between N1 habituation and high-order cognitive functions, it is reasonable to raise another interesting question of whether short-term auditory habituation is influenced by the processing of different types of phonological information in spoken-word sounds. A recent study directly addressed this issue and found a lexicality effect on N1 habituation to Mandarin spoken word-forms [25]. In this study, Yue and colleagues hypothesised differential patterns of the N1 habituation between the repetitions of a real Mandarin word (/ma1/) and those of a pseudoword (*/na1/), because they carry contrastive phonological information of lexicality. To be specific, a real word refers to a meaningful lexical unit in a language, whereas a pseudoword is a word-like speech form which obeys the phonological rules of this language (e.g., *bite* versus pseudoword *bipe in English, *cf*. [30,31]). In psycholinguistic studies, it is a common assumption that real words do not only correspond to sublexical-level representations for bottom–up processing (e.g., phonemes) but also to more abstract, lexical-level representations of whole words. In contrast, pseudowords do not have whole-word representations but are only mapped onto sublexical representations during speech recognition (e.g., [32,33]). Hence, a comparison between real words and pseudowords is usually carried out to separate the processing of lexical phonology from the sublexical processing, and thus, it reveals lexicality effects (e.g., [34,35,36,37]).

Taking advantage of this phonological contrast, Yue and colleagues (2017) [25] observed the degrees of N1 habituation throughout four repeated presentations (noted as S2–S5) of two word-forms, namely a real word (/ma1/) and a pseudoword (*/na1/). They found greater decrement of the N1 peak-amplitudes through the repetitions of the pseudoword than the real word in a right-hemispheric, fronto-central scalp region. After controlling the potential confound (e.g., the phonetic contrast between/m/ versus /n/), this lexicality effect of N1 habituation was explained by a manifestation of N1 habituation modulated by the processing of phonological information at a word level.

However, the study of Yue et al. (2017) [25] has some limitations. First, methodologically speaking, it only employed one word-form in each condition, and thus, the extremely low ecological validity might lead to some effect that is specific only for the two stimuli. Therefore, more evidence is needed to test for the hypothesis of the phonological tuning on N1 habituation.

Second, Yue et al. (2017) [25] did not investigate types of phonological information other than lexicality. This concern is of interest because previous EEG and MEG studies on early, pre-attentive speech perception have suggested that different kinds of phonological information such as lexicality and frequency of a spoken word can be processed automatically in the human brain, in time windows which, at least, partially overlap with the N1 time windows (e.g., [37,38]). For example, using mismatch negativity (MMN) as an indicator, researchers have revealed higher amplitudes of MMN to real words than to pseudowords (e.g., [37,39]; see [40] for an experiment with monosyllabic Mandarin word-forms). Moreover, some studies also found MMN evidence of early processing of lexical frequency, that is, high-frequency words tended to elicit stronger MMN responses than low-frequency words ([38,41]).

Furthermore, it was found that the processing of lexicality and frequency of a spoken word-form may even interact with each other during pre-attentive speech processing ([41,42]). For example, in an event-related potential (ERP) study, Silva and colleagues (2019) [42] found that only pseudowords that were generated based on low-frequency words elicited less negative N1-P2 responses than the real-word baseline, but the N1-P2 responses to pseudowords based on high-frequency words did not differ from the same baseline.

### 2.4. The Present Study

Putting these previous studies together, we aim to further test the hypothesis that N1 habituation is modulated by phonological information. In order to elicit decreased (habituated) N1, we adopt a short-term habituation design with Mandarin materials. To overcome the limitations in the previous study [25], monosyllabic Mandarin word-forms are generated by combining two types of phonological information, namely lexicality and usage frequency, factorially, and developed into four conditions, each of which consists of multiple types of word-forms.

Monosyllabic words in Chinese are basic lexical units which can either function as words or morphemes (for building up polysyllabic words). It is usually comprised of an onset (initial consonant), a rime (a vowel or a nasalised vowel with/n/or/ŋ/), and a tone [43]. Taking advantage of the special phonological system of Mandarin words, the lexicality of a word-form is manipulated by combining the same segmental template (i.e., onset + rime) with different tones, yielding existent lexical units (e.g., /tun1/(吞),/tun/+Tone1, means to swallow) or meaningless, pseudo word-forms (e.g., */tun3/,/tun/+Tone3) (see [44] for the investigations of the distinctions between the two types of word-forms with behavioural and electrophysiological measures). The same segmental templates shared by real and pseudo word-forms ensure that a potential lexicality effect does not involve any processing of anomalous segments (e.g., rime or onset), which cannot be avoided by using pseudowords in non-tone languages, such as English and French, which are derived through replacing phonemes in real words (*cf.*, [33,45]).

Meanwhile, the usage frequency of a word-form is manipulated as another phonological variable, which is measured with phonological frequency and computed by adding the total frequency of all possible words or morphemes sharing the same segment-tone pattern (*cf.* [46]). A monosyllabic Chinese word-form is known to have a number of homophones, sharing the same segment-tone pattern. For example, the word-form/yi4/has homophones such as “义” (righteousness), “意” (meaning), “亿” (a hundred million), etc. Therefore, it is reasonable to assume that when a spoken word-form is presented in an isolated way without any contextual information, the neural responses to the phonological part of an auditory word-form should be the function of one’s experience with all possible lexical tokens carrying the shared segment-tone template, as no contextual information can be used to disambiguate them. This assumption makes phonological frequency of a word-form a more suitable and practical measure for the current study than lexical frequency, because lexical frequency only quantifies the probability of a specific word to be used in a language, regardless of its potential homophones. Furthermore, it can be assumed that the perception of a pseudoword is determined by one’s exposure to real words with shared segmental templates, and thus, the pseudo word-forms inherit the phonological frequencies from the real words whose tones are changed to derive them (*cf*. [47,48]).

The effects of lexicality and usage frequency are examined to test the hypothesised phonological modulation on N1 habituation. If the two types of phonological information are independent modulators, only the main effects of lexicality and/or frequency should be observed. If they interplay with each other in modulating N1 habituation, an interaction between the two factors should be identified. Otherwise, if the two factors play no role in influencing N1 habituation to repeated spoken word-forms, no effects of the phonological information should be found, indicating that N1 habituation is a *pure* neural refractory process and does not allow any influence from the cognitive (i.e., phonological) processing of auditory input.

## 3. Materials and Methods

### 3.1. Participants

Thirty right-handed (adapted Edinburgh Handedness Inventory [49]) native Mandarin speaking participants (age mean = 21.7, *SD* = 4.4; 16 females), who reported no hearing or language disorders, were paid to participate in the study. They were all born and grown up in the northeastern region of China, where people are known to speak Mandarin. They were randomly assigned to one of the two lists of materials (see Appendix A). The experiment was approved by the Ethical Committee of School of Management of Harbin Institute of Technology. Informed consent was given before the experiment according to the Declaration of Helsinki.

### 3.2. Design and Materials

Spoken word-forms in four conditions were generated by combining the lexicality with two levels (high or low) and the usage frequency of the monosyllabic word-forms stimuli with two levels (real or pseudo word-forms). The four conditions are (1) high-frequency real word-forms (HPRW); (2) low-frequency real word-form (LPRW); (3) high-frequency pseudoword-form (HFPW); and (4) low-frequency pseudoword-form (LFPW).

Forty word-forms were derived from twenty segmental templates by combining one of which with two different tones, yielding a real and a pseudoword-form, respectively (e.g., /gei3/‘给’ (to give) and */gei1/). The frequency data of the word-forms were gleaned from the Chinese Internet Word Frequency List from the Lancaster Corpus of Mandarin Chinese [50]. The usage frequency of a real word-form is calculated by adding the frequencies of all its homophones. The frequency of a pseudo word-form is indicated by the phonological frequency of its base word-form. Segment-tone patterns that appear more than 200 times per million words were considered to be of high frequency and otherwise, they were considered to be of low frequency (*cf*. [51,52]).

Moreover, considering that tone regularity is a factor that influences spoken word recognition [53,54], all real word-forms included in this experiment were with a high tone regularity, meaning that a chosen word-form is the most regular combination of a segment template and a tone. For instance, [mai3] (to sell) was chosen because it is more regular than other segment–tone combinations, such as [mai2] (to burry), even though they are both meaningful word-forms. The control of tone regularity also ensured that the phonological frequency (i.e., usage frequency) of a real word-form is representative enough for its tone-manipulated pseudo counterpart. To avoid presenting the same segments in more than one condition to the same participant, materials were distributed into two lists following the Latin Square method. In addition, each list also contained 10 real and 10 pseudo word-forms as foils. The stimuli were previously recorded for a series of lexical-decision experiments by using high-quality recording equipment articulated by a female, native Mandarin speaker [44]. Stimuli were normalised for the same average intensity (75 dB) and duration (450 ms) by using an acoustic software programme, PRAAT [55].

**Figure 1:**
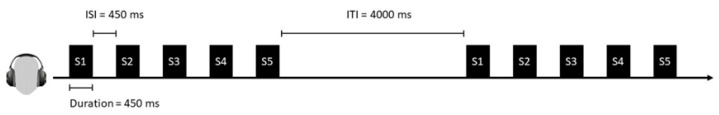
A demonstration of the short-term habituation paradigm.

A short-term habituation paradigm was employed to acquire decreased N1 responses. A stimulus was programmed to be repeatedly presented in trains, each of which held five presentation positions (S1 to S5), which were separated with a constant inter-stimulus interval (ISI) for 450 ms. Given an inter-train interval (ITI) for 4 s with a 200 ms jitter, the electrophysiological response to a spoken word-form could be expected to recover from the short-term habituation of N1 in the previous train, according to many previous studies [12,18,25]. Figure 1 presents how two trains are delivered in the current design.

The word-forms in each list were divided into five blocks in which one word-form out of five for each condition only appeared in one block. In a block, a train carrying the same word-form was delivered 11 times. Four foil word-forms were included and varied for each block but kept the same across the two lists. A train of a foil word-form was presented five times in a block. The trains of stimuli were pseudo-randomly presented in a way that those of the same condition were presented no more than three times in a row. A 1.5 min stimulus-free break was set between every two blocks.

### 3.3. EEG Data Acquisition

The EEG of each participant was recorded in a sound attenuated cabin with constant, dim lightness, seated in front of a PC monitor placed at a distance of about 1.2 m. They were randomly administered to be exposed to one stimulus list and were suggested to refrain from unnecessary body movement. During the EEG recording, auditory stimuli were delivered binaurally and passively via a pair of Sennheiser headphones. To avoid that the habituation of AEPs is potentially confounded with selective attention [26], participants were instructed to watch a silent cartoon movie without subtitles, and asked to remember the contents for a movie comprehension test which was administered after the EEG acquisition. The comprehension test required participants to read 12 statements about the movie and judge whether they matched the contents of the movie by choosing either “Yes”, “No”, or “Cannot remember”. Moreover, they were encouraged to ignore any sounds that would be presented from the headphones.

The EEG signal was recorded by a LiveAmp amplifier (Brain Products) via 32 Ag/AgCl electrodes situated on an elastic cap, according to the extended international 10-20 system, with a 500 Hz sampling rate. One electrode was placed at the right infraorbital ridge to monitor ocular movement. The online reference was FCz and the ground electrode was AFz. The impedance of electrodes was kept below 5 kΩ.

### 3.4. EEG Data Analysis

The offline processing of the EEG data was performed in Brain Vision Analyzer 2.0 (Brain Products). A band-pass filter between 1 and 30 Hz was first applied, and then, the filtered data were re-referenced to the average amplitudes recorded from the two mastoids. Voltage levels exceeding ±40 *μ*v in any channel were rejected as artefacts. Then, the surviving EEG recording was segmented separately for the five presentations (i.e., S1–S5) of each stimulus type, with an epoch of 600 ms, between −100 ms before and 500 ms after the stimulus onset. Baseline was corrected according to the pre-stimulation responses (−100 to 0 ms).

Peak amplitudes of N1 were quantified with a peak-to-peak measurement by calculating the amplitude difference between an N1 peak and the peak of its preceding prominent positive component P1 (*cf*. [56,57]). For this measurement, the peak amplitudes of P1 and N1 were detected in two time-windows, respectively: 40–100 ms (P1) and 80–170 ms (N1) for each type of stimuli at every presentation position, per participant. The time windows were defined by referring to previous studies (e.g., [58,59]) and adjusted according to visual inspection of the data. The degree of N1 habituation at each presentation position was quantified by a habituation index, which is calculated as the ratio of the N1 peak amplitude in a repeated position (S2–S5) to that in the initial presentation (S1) for each of the four stimulus types. Accordingly, the greater the index is, the less the relative auditory habituation takes place. The formula of its calculation is:

Habituation index = N1S_n_/N1S_1_ (n = 2, 3, 4, 5)

The N1 habituation was analysed at two representative electrodes, namely C3 and C4. The selection of the two electrodes were based on three criteria, which were defined *a priori*. First, they should be around the vertex electrode Cz to reflect typical short-term auditory habituation [18]. Second, electrodes from the right fronto-central scalp region should be included to make the current results comparable with the phonological effect on N1 habituation reported in Yue et al. (2017) [25]. Third, electrodes from the left fronto-central area should be chosen as they usually capture pre-attentive neural processing of speech [60]. Putting these together, we selected C3 (left hemispheric) and C4 (right hemispheric) to investigate the hypothesised modulation of phonology on N1 habituation. Moreover, at the two electrodes, artifact-free epochs of AEP data accounted for 94% of the total of number of trials.

The data analysis began by testing the validity of the current paradigm in eliciting reliable N1 habituation. A 5 × 2 × 2 × 2 repeated measures ANOVA was first conducted to examine whether the N1 responses at S1 differ from the other repeated presentations with four factors: PRESENTATION (S1 ~ S5), LEXICALITY (Real vs. pseudo word-forms), FREQUENCY (high versus low frequency), and ELECTRODE (C3, C4). A main effect of PRSENTATION would verify decreased N1 responses through stimulus repetitions.

Further inferential statistical analyses were conducted with the habituation-index data at four repetition positions, separately. To deal with some outliers (i.e., extremely high or low habituation indices), those falling out of ±1.5 interquartile range (IQR) were replaced by the farthest values within the range at each electrode per condition (replacement rate is 6.6%). After dealing with the outliers, the analysis of phonological effects on habituation began with a 2 × 2 × 2 repeated measures ANOVA with three factors: LEXICALITY, FREQUENCY, and ELECTRODE. Repetition position was not considered as a factor because the degree of N1 decrement caused by habituation may not be a linear function of presentation positions [13,61], and thus, presentation position (i.e., PRESENTATION) may not be a suitable factor for ANOVA. If any interactions between LEXICALITY and FREQUENCY could be identified, further analyses would be carried out to check how the factors lead to differential habituation patterns in the four types of stimuli. Greenhouse–Geisser correction was applied to adjust the degrees of freedom for the *F* tests when the sphericity assumption was violated according to Mauchly’s test. Bonferroni corrections were performed when appropriate. The uncorrected degrees of freedom and the adjusted *p*-values were reported. The significance criterion was *p* < 0.05.

## 4. Results

**Figure 2:**
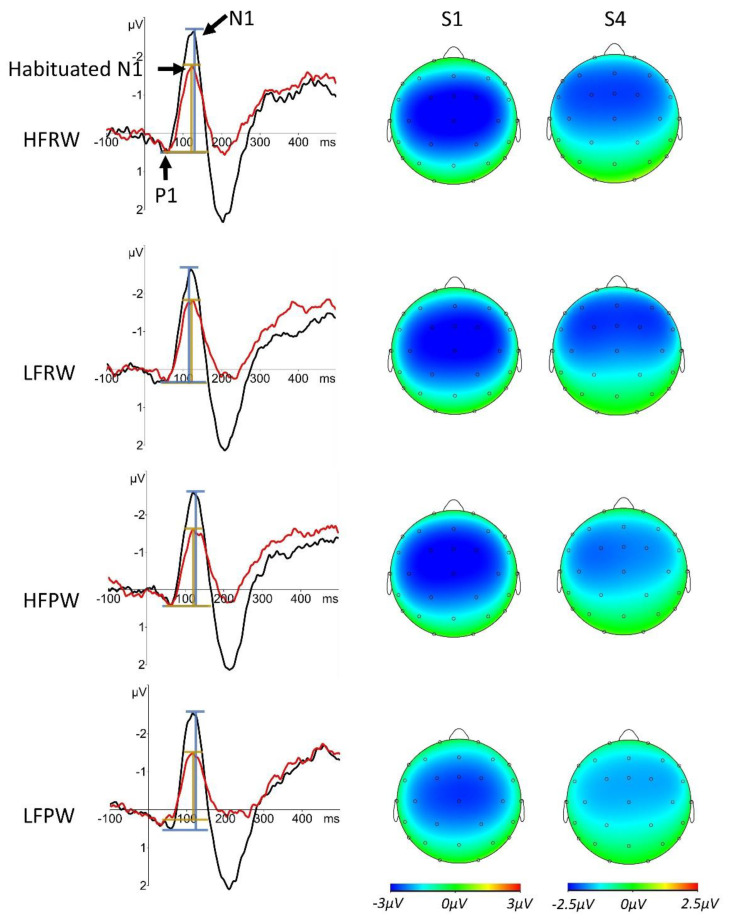
The grand average of the averaged AEP waveforms of C3 and C4 (left column) for the four conditions in S1 (black) and S4 (red), aligned with the topographic maps of the grand-averaged N1 according to the peak latency at C4 in S1 (middle column) and S4 (right column), in the four conditions. Differential scales were used for S1 and S4 for a demonstrative purpose. In the AEP waves, the blue bars denote the N1 peak-to-peak amplitudes (N1-P1) in S1, compared with the habituated N1, as marked by the golden bars. Note the relatively lower ratio of the length of the line segment for S4 in the length of the segment for S1 in low-frequency pseudo word-forms (LFPW) than in high-frequency pseudo word-forms (HFPW) and in low-frequency real word-forms (LFRW), respectively.

**Figure 3:**
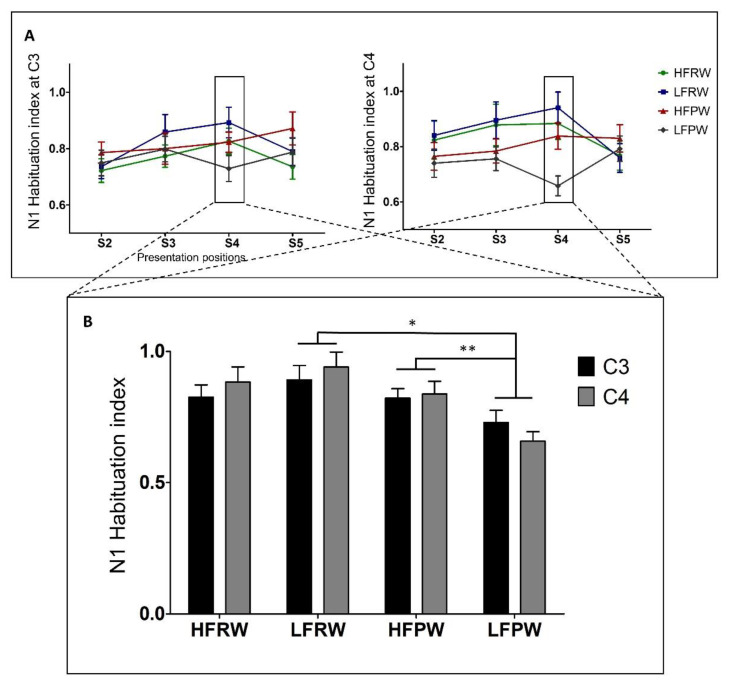
The phonological modulation effects of N1 habituation. (Panel **A**) demonstrates the N1 habituation indexes in the four conditions, in each stimulation position, at C3 and C4. (Panel **B**) depicts the N1 habituation indices in S4 and the significant interactive effects between lexicality and usage frequency. The error bars represent the standard error of the mean (SEM), * *p* < 0.05, ** *p* < 0.01.

Short-term N1 habituation was successfully obtained with the current paradigm as reflected by the apparent decrements of the N1 responses in repeated stimuli (S2–S5) relative to S1 from visual inspection (see Table 1, also see Figure 2; Figure 3 for demonstrations of the decreased (habituated) amplitudes of the cortical AEP components, especially the N1 component, in S4 relative to those in S1 and the N1 topography in S1 and S4). This response pattern is confirmed by a significant main effect of PRESENTATION (*F* (4, 116) = 32.55, *p* < 0.001). This result attests the validity of the current short-term habituation design. Moreover, the participants had very high accuracy rates in the statement judgement task (*M* = 90.4%, *SD* = 7.1%), suggesting that they have focused on the movie-watching task, and therefore, can be assumed to spare little attention on the auditory stimuli. A summarisation of the N1 peak amplitudes and the habituation indices can be seen in Table 1.

The inferential statistical analyses with the habituation-index data first revealed a main effect of LEXCIALITY (*F*(1, 29) = 9.249, *p* = 0.02) and a significant interaction between LEXICALITY and FREQUENCY (*F*(1, 29) = 10.018, *p* = 0.016) only in S4, after Bonferroni corrections were performed (Figure 3). Following this interaction, a main effect of LEXICALITY was found for low-frequency real and pseudo word-forms (*F*(1, 29) = 16.854, *p* < 0.001), as well as an interaction between LEXICALITY and ELECTRODE (*F*(1, 29) = 5.419, *p* = 0.027). Further analyses revealed two main effects of LEXICALITY at both C3 (*F*(1, 29) = 6.762, *p* = 0.015) and C4 (*F*(1, 29) = 24.432, *p* < 0.0001). These results suggest that the degree of N1 habituation in low-frequency pseudo word-forms around the vertex is greater relative to that in low-frequency real word-forms. The effect size in C3 is about 16% and that in C4 is 28% (C3: LFPW: *M* = 0.73, *SD* = 0.25, LFRW: *M* = 0.89, *SD* = 0.30; C4: LFPW: *M* = 0.66, *SD* = 0.20, LFRW: *M* = 0.94, *SD* = 0.31) (Table 1, Figure 2 and Figure 3).

Furthermore, unpacking the interaction between LEXICALITY and FREQUENCY also yielded a main effect of FREQUENCY for the two conditions of pseudo word-forms (*F*(1, 29) = 9.793, *p* = 0.004). This main effect confirms that the degree of N1 habituation in low-frequency pseudo word-forms is about 9% greater than in high-frequency pseudo word-forms at C3 (LFPW: *M* = 0.73, *SD* = 0.25; HFPW: *M* = 0.82, *SD* = 0.20) and 18% greater at C4 (LFPW: *M* = 0.66, *SD* = 0.20; HFPW: *M* = 0.84, *SD* = 0.26) (Figure 2 and Figure 3).

*Post hoc* analyses were conducted to test for the possibility that the phonological effects on N1 habituation at S4 position were just a reflection of some processing effects on the P1, N1, and/or N1-P1 responses alone at S4 or S1 position, the initial presentation in a train. To this end, repeated measures ANOVA was conducted on the peak amplitudes of P1 and N1 component (baseline-to-peak measurement), and the peak-to-peak amplitudes of N1-P1, which were used to compute the habituation indices, with three factors: LEXICALITY, FREQUENCY, and ELECTRODE, at S1 and S4, respectively. Neither main effects of LEXICALITY nor an interaction between LEXICALITY and FREQUENCY were found in the analysis with the peak amplitudes at the two positions. These results suggest that the phonological effects on N1 habituation reported in this study may not directly reflect the auditory processing of spoken words but could be decided by the modulatory roles of the phonological information in tuning the process of habituation.

## 5. Discussion

Although it is common to observe that repetitively presented auditory stimuli elicit decreased amplitudes of the N1 component of the AEP, less is known about the role of cognitive processes of the stimuli in influencing this physiological phenomenon. To address this issue, we hypothesised modulatory roles of two kinds of phonological information of spoken word-forms, namely lexicality and usage frequency. Our data first confirm that the current design could elicit a short-term habituation of auditory N1 throughout repetitions of spoken word-form stimuli. Then, we found evidence that the two types of phonological information are indeed factors that modulate the N1 habituation in an interactive way, which is supportive of our phonological modulation hypothesis. More specifically, our data reveal that at the two electrodes of interest (C3 and C4), the N1 habituation to low-frequency pseudo word-forms is greater than that to low-frequency real word-forms (lexicality effect) and high-frequency pseudo word-forms (usage-frequency effect), respectively. These effects are identified at S4 position in the trains.

Additionally, the results of the *post hoc* analyses of the peak amplitudes of N1 and its preceding positive P1 component at either S1 or S4 ensure that the interactive effects of phonology on N1 habituation are not just a reflection of the phonological processing itself. Rather, they suggest the modulatory effects of phonological processing on N1 habituation. Therefore, to the best of our knowledge, our finding is the first showing that the short-term habituation of auditory N1 can be interactively modulated by different types of phonological information of spoken word-forms.

### 5.1. The Effects of Lexicality and Usage Frequency

Here, the lexicality effect of N1 habituation is partially in line with a previous study [25]. In that study, researchers found a greater habituation of auditory N1 through the repetitions of a pseudoword than a real word in Mandarin over a right-hemispheric region of interest, which is composed by three electrodes of F4, FC4 and C4. In the current study, we adopted a similar experimental design but using multiple real and pseudo word-forms, and we observed a lexicality effect in both of the two electrodes of interest at only one presentation position (i.e., S4) by comparing the N1 habituations to real and pseudo word-forms with low usage frequencies.

Notably, there are some distinctions between the two lexicality effects, in terms of their scalp distributions and the presentation positions, in which the phonological effects were found. We cannot find readily explanations for these distinctions because of the poor literature on this topic, but we would ascribe them to the methodologies employed for the elicitation of N1 habituation and data analysis instead of treating them as indicators of totally different habituation mechanisms. For example, differing from Yue et al. (2017) [25], in the current study, we exploited multiple types of word-forms for each condition generated based on combinations of two phonological factors. By presenting the stimuli for each condition in the same number of trains as Yue et al., 2017 [25] (i.e., [57]), we created a habituation environment with much higher phonological variability and richness, which are known to impact speech perception [62,63] and acquisition [64,65]. Consequently, the repetitions of speech stimuli might not undergo exactly the same neural adaptation when different numbers of the types of word-forms are used, leading to phonological effects in both the right and left hemispheric electrodes in the current study but only in the right hemispheric electrodes in Yue et al. (2017) [25].

With respect to the presentation–position–specificity (S4) of the phonological effects in our study, we see the reason as the different statistical approaches applied in different studies [25]. When analysing the lexicality effects on habituation, Yue et al. (2017) [25] treated repeated presentation positions, namely S2–S5, as one factor with four levels. Contrastively, in the present study, we considered that N1 habituation could be a non-linearity function of presentation position within a train [13], and thus, we performed analyses for each position, separately, with Bonferroni corrections. As a result, Yue et al. (2017) [25] might have failed to locate effects at some specific position, as the potentially non-linear feature of habituation at different positions prevented the researchers from observing an interaction between presentation and other linguistic variables.

In spite of these differences, both lexicality effects on N1 habituation in the two studies are clear evidence that phonological information indeed tunes N1 habituation to spoken word-forms. More importantly, beyond the lexicality effect, our results further suggest a role of the usage frequency of a spoken word-form in influencing N1 habituation, which interacts with lexicality. Specifically, the lexicality effect on N1 habituation is only observed in low frequency word-forms; in the meanwhile, a usage-frequency effect on N1 habituation only emerges in pseudo word-forms. These findings indicate that different types of phonological information may not be independent modulators (i.e., lexicality and usage frequency) but affect N1 habituation through interacting with each other.

These data are, first, coherent with previous neurophysiological studies, finding an automatic processing of phonological information such as lexicality [37,39] and lexical frequency [66] in pre-attentive speech perception. Then, the interaction between these phonological factors is also in line with a few previous studies, revealing an interplay between lexicality and lexical frequency during speech perception [38,42]. More interestingly, in those studies, low-frequency pseudowords were usually found to elicit lower amplitudes of early AEP responses relative to the real word or the high-frequency pseudoword baseline, which are very similar to the N1 habituation patterns that we found in the current study.

Despite these similarities, it must be noted that the phonological effects as we discuss here are not equal to the processing of phonological information *per se*. It is first because we only found phonological tuning on N1 habituation but did not observe any phonological effects in the N1, P1, and N1-P1 responses at the positions (i.e., S1 and S4) where the habituation indices were calculated and revealed the phonological modulation. The other reason is that the latency of auditory N1 (about 100 ms post-stimulus onset) is indeed too short for the unfolding of all necessary phonological information for recognising a word-form (e.g., lexicality and frequency), and thus, it is unlikely that the phonological effects on N1 habituation are direct reflectors of the processing of phonological information.

In addition, the phonological effects in the current study are not likely to be caused by confounders associated with phonetic contrasts—the tonal contrasts between real and pseudo word-forms with the same segmental templates (e.g., /mai3/and */mai1/) or the segmental differences between high- and low-frequency word-forms (e.g., /mai3/versus/niang2/). Admittedly, there are studies reporting neurophysiological responses related to the pre-attentive processing of tonal and segmental features in Chinese language speakers (e.g., [67,68,69]). However, such accounts are unlikely to be applicable for the current results because of the following reasons.

First, the phonological effects here were hypothesised *a priori* based on previous studies (e.g., [10,25]). Particularly, the lexicality effect in the current study is partially coherent with previous literature in which the effect was obtained by using a real (/ma1/) and a pseudoword-form (/na1/), carrying the same tone but different initial onset consonants. In that study, no effects were found between the two control words for the contrastive onsets (/mi2/versus/ni2/), which is indirect evidence that the effect on N1 habituation is originated from the phonological processing rather than phonetic processing.

Second, to date, the number of studies showing short-term auditory habituation’s sensitivity to phonetic cues remains scarce. Many previous studies only revealed differential habituation patterns between speech and non-speech (could be speech-like) sounds, which were not in the phonetic domain neither [10,14,24]. Admittedly, some previous studies reported that repetitions of different speech forms could elicit differential blood oxygen level-dependent (BOLD) responses as measured with fMRI [36,70,71].

However, we are reluctant to directly transfer these findings to explaining our data. It is first because the experimental paradigms varied greatly in different studies. As a result, it cannot be certain that their data reflect the same habituation mechanism, precisely auditory short-term habituation, as the current study. The other concern is that the acquisition of BOLD responses to stimuli usually requires more than 1 s [72], which is much longer than the instant recording of neurophysiology. Therefore, the phonetic effect on reduced BOLD responses may not correspond to the habituation of auditory N1 whose latency is as early as about 100 ms (0.1 s) post-stimulus onset.

Third, the design of the current experiment made a phonetic account less likely to be suitable for our data. Although the distributions of tones and segments in different conditions were not meant to be completely balanced, they were generally comparable, as can be seen from the materials (Appendix A). More importantly, since the participants were only presented with one list of the materials (Latin square method), the lexicality and usage frequency effects were obtained by comparing phonologically unrelated materials. Therefore, it can be assumed that the phonetic contrasts between different conditions are just a random noise, which could be averaged out by applying multiple word-forms. This design further minimises the chance that our phonological modulation effects are merely caused by some phonetic factors. Therefore, we believe our design allows us to probe the phonological modulation hypothesis before there is convincing evidence of phonetic modulation of auditory N1 to speech sounds. Nonetheless, investigations on the phonological effects can be conducted by controlling the tonal or the segmental factors in the future.

### 5.2. Theoretical Implications

After discussing the phonological effects on N1 habituation, the next issue that we want to further elaborate upon is how these effects provide insights into the auditory short-term habituation and its interface with the phonological system. First of all, the interaction between phonological factors clearly suggests that N1 habituation is not likely to be a totally bottom–up refractory process. Otherwise, no systematic differences should be observed during perceiving spoken word-forms that are acoustically similar but phonologically distinctive (for a similar conclusion, see [24]). However, we want to emphasise that we are not meant to refute the existence of refractoriness which could be a basic mechanism underlying neural habituation induced by rapidly repeated auditory stimuli [23] but rather to highlight the impact of phonological processing on this mechanism.

Second, the phonological modulation on N1 habituation is unlikely to be caused by general cognitive functions, such as expectation [29] and selective attention [26], which are not specific to linguistic processing. This is because the participants were passively delivered with stimuli in different conditions by means of trains that consisted of the same number of presentation positions, which were separated by a short, constant interstimulus interval. Therefore, even if the participants had some expectations after being familiar with the experimental settings, such an expectation should be equally applied for the N1 habituation in all conditions, resulting in no differences between conditions. However, this pattern is apparently not what we observed in the data. Furthermore, the participants’ attention was successfully distracted by a task which is unrelated to hearing. This is a common practice when the automatic cognitive processing of speech sounds outside the focus of attention is studied [73]. Hence, it can be assumed that the participants were in a stable attentional status during the experiment, and thus, the cognitive functions are unlikely to explain the phonological effects on N1 habituation.

**Figure 4:**
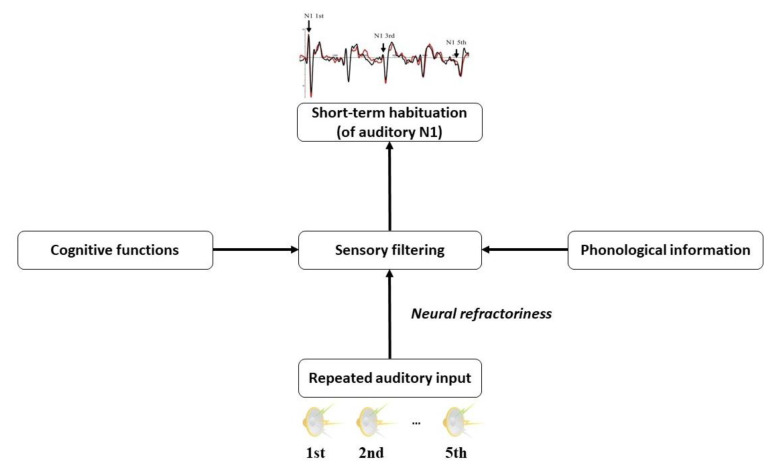
Demonstration of the cognitive factors’ modulation on N1 habituation via sensory filtering induced by repeated auditory stimuli. When cognitive factors are not involved in the perception of repeated auditory stimuli, N1 habituation may be subjected to neural refractoriness. However, the processing of cognitive features of the input can activate the modulators in sensory filtering which mediates between cognitive processing and bottom–up *pure* physiological mechanism.

After excluding the possibility of the refractory and cognitive views, the most likely account for the reliable interaction between lexicality and usage frequency in the N1 habituation data could be that the phonological information are modulators of N1 habituation to spoken word-forms. How could this modulation take place? Here, we tentatively propose sensory filtering as a candidate mediator between the short-term habituation of N1 and the processing of phonological information, which tunes the magnitude of N1 habituation according to phonological input (see Figure 4). Sensory filtering is considered as an automatic brain function that filters out irrelevant information to protect the sensory system from being overloaded by repeated input [27]. Abnormal sensory filtering has been indexed by the evoked P1/N1 responses or their attenuation in patients with psychiatric [74], neurological [75], genetic diseases [76], as well as in children with autism spectrum disorder [27].

Accordingly, the relevance of a sensory input is automatically appraised for whether and to what extent repeated inputs to be filtered. From this regard, in the current study, low-frequency pseudo word-forms are no doubt the least possible sound patterns of Mandarin speech, and therefore, they are very likely to be judged as “irrelevant” speech inputs compared with low-frequency real word-forms and high-frequency pseudo word-forms. Then, it is reasonable to observe the phonological effects on the N1 habituation, that is, more habituated N1 responses through the repetitions of the “irrelevant” speech sounds than other types of word-forms.

This sensory perspective also helps to explain why phonological modulation occurs so early when only a very small portion of a spoken word-form is unfolded. For Mandarin, the identification of an isolated monosyllabic word requires about 160 ms from the onset of a word [77], which is later than a typical N1 time window when the phonological modulatory effects have already emerged. A possible explanation is that throughout the repetitions of the same stimulus in a train, automatic analysis of the phonological information of the stimulus might have been stored in the transient sensory memory, which further supports sensory filtering. This explanation follows a similar logic of previous studies on the relationship between the sensory memory and the high-order cognitive functions (*cf.* [78,79]).

Furthermore, this sensory-filtering idea can also shed some light on previous studies that found less attenuated N1 responses to speech sounds relative to non-speech sounds [10,24]. It is possibly because the speech sounds apparently offer more information than non-speech sounds, and are thus appraised as more “relevant” stimuli (see [14] for an exception). Figure 4 demonstrates a tentative schema of how repeated auditory inputs induce short-term habituation which is modulated by phonological and cognitive factors via sensory filtering.

### 5.3. Limitations and Future Research

The current study has some limitations which merit future studies. Methodologically speaking, the current design only adopted trains of five with a fixed inter-stimulus interval, and only 55 trains (trials) for each condition were presented. Consequently, there is no chance to examine how the modulatory effects are influence by experimental settings. For instance, in the current study, the interactive phonological modulation on N1 habituation was found at the fourth presentation position. It would be very interesting to investigate if this position, where phonological effects are seen, changes when a shorter or a longer ISI is used or when longer or shorter trains are administered [10,24], given potential altered recovered cycles of short-term habituation to spoken word-forms (*cf.* [9,23,80]). In addition, despite the fact that the sample size from the current study falls within the upper end of previously published studies (e.g., 7 in [10], 19 in [24], 30 in [25]), future replications with larger sample size are nevertheless necessary.

Another possible methodological limitation is that by using multiple word-forms for a condition, we did not control phonetic, semantic and syntactic variables which have been shown to influence pre-attentive speech processing (see [60] for a review). This is due to several reasons: first, since our focus is on phonological-level effects, we applied a design that might make other possible effects become random noise through the repeated presentations of multiple phonologically-unrelated word-forms. Second, due to the small number of previous studies on the cognitive (especially phonological) modulation on N1 habituation, there are few data showing that N1 habituation to spoken words is impacted by these psycholinguistic factors yet. However, it would be worthwhile for studies in the future to investigate whether N1 habituation is sensitive to a broader range of psycholinguistic factors.

Additionally, so far, all evidence of phonological modulation on N1 habituation comes from monosyllabic Mandarin word-forms. Therefore, this mechanism needs more tests by using polysyllabic Mandarin word-forms or materials from other languages, especially Indo-European languages, most of which do not rely on lexical prosody (e.g., tone, stress) to distinguish the meanings of words with identical segments.

Theoretically speaking, although our sensory-filtering account offers an explanation for the relatively greater habituation in the most “irrelevant” word-forms (i.e., low-frequency pseudo word-forms) than the other two more “relevant” types of word-forms, our data cannot provide more information about how the “relevance” (or “irrelevance”) of speech sounds is judged.

A possible source of the judgment of relevance may rest on the automatic neural decoding of speech sounds. According to speech perception theories, speech input is decoded by being mapped onto previously stored neural representations at a remarkable pace [81,82]. Such representations could be roughly divided into lexical and sublexical levels [83], which may interact with each other for a high efficiency of processing (e.g., the TRACE model [84] and a recent model following the predictive coding principles [85]). Accordingly, the pre-attentive processing of phonological information such as lexicality and frequency may elicit specific neural signatures by different spoken word-forms which are used to originate an appraisal of relevance or irrelevance. However, the detail of this process should be very carefully studied in the future, as we did not find robust habituation effects in the comparisons between high-frequency real words and other types of word-forms in the current study, suggesting complicated modulatory mechanisms on N1 habituation when the relevance of a word-form is considered.

## 6. Conclusions

An emerging number of studies have suggested that the short-term habituation of AEPs may be tuned by the cognitive processing of auditory stimuli. In the present study, we further hypothesise phonological modulation on the short-term habituation of the auditory N1. To test for this hypothesis, we investigate the potentially modulatory roles of two kinds of phonological factors: lexicality and usage frequency of spoken word-forms, each of which is divided into two levels. The orthogonal combinations of the two factors yield four conditions. By quantifying the N1 habituation to the multiple Mandarin monosyllabic word-forms in each condition, we find more habituated N1 to low-frequency pseudo word-forms than that to low-frequency real word-forms and high-frequency pseudo word-forms, respectively. This is the first evidence that the two phonological factors interactively modulate N1 habituation. Based on this finding, we propose sensory filtering as a candidate mechanism that mediates the processing of phonological information and short-term auditory habituation.

## Figures and Tables

**Table 1:** Peak-to-peak amplitudes of the N1 responses (i.e., measured as N1-P1) at S1, and the N1 habituation index for the four repeated positions in the two electrodes of interest (C3 and C4).

Condition	Electrode	Amplitude S1 (*μ*v)		Habituation Index S2		Habituation Index S3		Habituation Index S4		Habituation Index S5	
		M	SD	M	SD	M	SD	M	SD	M	SD
HFRW	C3	4.38	1.65	0.72	0.23	0.77	0.22	0.83	0.25	0.74	0.24
	C4	4.05	1.66	0.82	0.37	0.88	0.41	0.88	0.32	0.77	0.29
LFRW	C3	4.19	1.83	0.74	0.23	0.86	0.34	0.89	0.30	0.79	0.27
	C4	3.68	1.53	0.84	0.30	0.90	0.36	0.94	0.31	0.76	0.29
HFPW	C3	4.18	1.61	0.79	0.20	0.80	0.31	0.82	0.20	0.87	0.32
	C4	4.10	1.66	0.76	0.27	0.78	0.24	0.84	0.26	0.83	0.27
LFPW	C3	4.32	1.59	0.75	0.25	0.80	0.25	0.73	0.25	0.79	0.27
	C4	4.20	1.64	0.74	0.28	0.76	0.23	0.66	0.20	0.79	0.25

A higher habituation index means a smaller ratio of the decrement of the N1 amplitude as compared with the N1 to the initial stimulus. HFRW: high-frequency real word-form; LFRW: low-frequency pseudoword-form; HFPW: high-frequency pseudoword-form; LFPW: low-frequency pseudoword-form.

## Data Availability

Research data will be provided by the corresponding author, J.Y., upon reasonable request.

## Appendix A

**Table A1:** The materials of the current experiment for the two lists presented separately.

List1				List2			
HFRW	HFPW	LFRW	LFPW	HFRW	HFPW	LFRW	LFPW
ran2	mai1	jiong3	niang3	mai3	ran4	niang2	jiong1
chun1	ru1	tun1	diu4	ru4	chun4	diu1	tun3
che1	kan2	mie4	nie3	kan4	che2	nie1	mie3
heng2	lun3	pie3	rao1	lun4	heng3	rao4	pie2
gei3	nü4	niao3	reng3	nü3	gei1	reng2	niao1
HFRP = high-frequency real word-form, HFPW = high-frequency pseudo word-form, LFRW = low-frequency real word-form, LFPW = low-frequency pseudo word-form.							
